# 

**Published:** 1976

**Authors:** 


JANUARY/APRIL 1976

VOL. 91 (i/ii) No. 337/8

